# Perpendicular Magnetization Behavior of Low- Temperature Ordered FePt Films with Insertion of Ag Nanolayers

**DOI:** 10.3390/ma9030209

**Published:** 2016-03-18

**Authors:** Da-Hua Wei

**Affiliations:** Institute of Manufacturing Technology and Department of Mechanical Engineering, National Taipei University of Technology (TAIPEI TECH), Taipei 10608, Taiwan; dhwei@ntut.edu.tw; Tel.: +886-2-2771-2171 (ext. 2022)

**Keywords:** perpendicular magnetization behavior, FePt, Ag, low temperature ordered, nanolayers

## Abstract

FePt-Ag nanocomposite films with large perpendicular magnetic anisotropy have been fabricated by alternate-atomic-layer electron beam evaporation onto MgO(100) substrates at the low temperature of 300 °C. Their magnetization behavior and microstructure have been studied. The surface topography was observed and varied from continuous to nanogranular microstructures with insertion of Ag nanolayers into Fe/Pt bilayer films. The measurement of angular-dependent coercivity showed a tendency of the domain-wall motion as a typical peak behavior shift toward more like a coherent Stoner-Wohlfarth rotation type with the insertion of Ag nanolayers into the FePt films. On the other hand, the inter-grain interaction was determined from a Kelly-Henkel plot. The FePt film without insertion of Ag nanolayers has a positive δ*M*, indicating strong exchange coupling between neighboring grains, whereas the FePt film with insertion of Ag nanolayers has a negative δ*M*, indicating that inter-grain exchange coupling is weaker, thus leading to the presence of dipole interaction in the FePt–Ag nanogranular films. The magnetic characteristic measurements confirmed that the perpendicular magnetization reversal behavior and related surface morphology of low-temperature-ordered FePt(001) nanogranular films can be systematically controlled by the insertion of Ag nanolayers into the FePt system for next generation magnetic storage medium applications.

## 1. Introduction

Nanogranular magnetic materials with a perpendicular magnetic easy axis have the most potential as candidates for application in ultrahigh-density magnetic storage media due to the fact that areal recording density of hard disk drives (HDDs) needs to be increased to meet the requirement to store exponentially increasing digital data information. The currently used Co-based compounds media do not have enough thermal stability to resist thermal fluctuation and demagnetization effects due to the material intrinsic property of their magnetocrystalline anisotropy constant (*K*_u_). In order to satisfy the future storage requirement, high-*K*_u_ materials have been considered as a possible solution that could maintain enough thermal stability not only to delay the superparamagnetic effect but also overcome thermal fluctuation [1,2]. The *L*1_0_-ordered FePt intermetallic phase with excellent material intrinsic properties such as high *K_u_* ~ 7 × 10^7^ erg/cm^3^, μ_0_*M_s_* ~ 1.43 T, and energy products’ *BH* values has undergone rapid development up to now due to its promising applications including monolithic microwave integrated circuits, spin electronic devices, and next generation ultrahigh-density magnetic information storage media [3,4,5,6,7,8,9,10,11]. However, the formation of the *L*1_0_-ordered FePt phase usually requires an elevated treatment of either a substrate heating or a post-annealing temperature higher than 500 °C, which also results in the large grain size distribution and is not favorable for industrial productions. The large grains with a strong inter-grain coupling interaction will cause high read/write noises in the recording medium. Many approaches have been reported to use single-crystal substrates or optimum buffer/seed layers in order to form (001) textured Fe-based compound phases with preferred out-of-plane direction with the magnetic anisotropy that is perpendicular to the film plane [12,13,14,15,16,17,18,19,20,21,22,23,24]. For example, Speliotis *et al.* used MgO/Cr bilayers as a buffer layer to fabricate a (001) textured FePt film on amorphous glass substrate that exhibited good perpendicular anisotropy [25]. The method of Fe/Pt multilayer growth also used in this work can effectively lower the ordering temperature of martensite-based phase transformation from face-centered-cubic (fcc-disordered) to face-centered-tetragonal (fct-ordered) state due to the faster Pt and Fe atoms to interdiffuse at the Fe/Pt interface to form a chemically *L*1_0_-FePt compound phase [26,27,28,29,30,31,32]. FePt alloy films added with Ag-top, Ag-under, and Ag-doped methods have been reported not only to improve the ordering phase transformation, but have also been employed as the seed of promoting the (001) preferred crystallographic orientation of FePt films [33,34,35,36,37,38,39,40,41]. The Ag element was also used to serve as a matrix phase to separate FePt grains due to immiscible Ag atoms from the FePt alloy. Even many works reported the microstructure modulation of the FePt-Ag system by the inactive atomic mobility of FePt reacted with Ag, but the corresponding evolution of the perpendicular magnetization reversal mechanism of the FePt-Ag nanocomposite structures is still deficient [42,43,44,45,46]. Due to the inactive atomic mobility of the FePt phase, the perfectly continuous film structures are usually formed at growth temperatures below 600 °C [47,48]. Thus, the high formation temperature is needed to achieve a granular structure of the ordered FePt with high coercivity, which is not suitable for industrial productions. The purpose of this work is to explore and control the exchange coupling interaction between the neighboring grains and formation of granular FePt films at a low substrate heating temperature without any post-annealing which is just formed by employing the insertion of Ag nanolayers. In this present article, a method for the fabrication of FePt nanogranular films with (001) crystallographic orientation along with the insertion of Ag nanolayers was employed to promote this self-organized nanogranular structure at a low process temperature of 300 °C. This work shows a significant performance for FePt films inserted with Ag nanolayers on the perpendicular magnetization behavior and microstructure control, and the corresponding intergranular exchange coupling and magnetization mechanism of the FeP–Ag nanocomposite films are also discussed.

## 2. Experiments and Multilayer Film Structures

FePt binary alloy films are composed of [Fe(0.5 nm)/Pt(0.5 nm)]_18_ repeated periodic structures with 18 bilayers, and this means that 0.5 nm layers of Fe and Pt were deposited alternately for a total nominal thickness of 18 nm onto MgO(100) single-crystal substrates without any buffer layer via an electron beam evaporation system under a vacuum of 2 × 10^−^^8^ Torr. The FePt–1Ag sample, after 9 nm of FePt, had an ultrathin 0.5 nm layer of Ag inserted, so there was a single 0.5 nm layer of Ag inside the 18 nm of FePt. The FePt–2Ag sample, after each 6 nm of FePt, had an ultrathin 0.5 nm layer of Ag inserted, so there were two Ag nanolayers inside the 18 nm of FePt. The FePt–Ag nanocomposite structures were formed and all the details are listed in Table 1. All multilayer film structures were evaporated at a low substrate heating temperature of 300 °C with an evaporation rate of around 0.01 nm/s. The crystallographic orientation and structural characterization of FePt films were evaluated by *ex situ* X-ray diffraction (XRD) with Cu *K*_α_ radiation. The surface morphology of the FePt films was observed by the field emission scanning electron microscope (FE-SEM), and all grain analyses were acquired and processed using the digital micrograph (DM) software. The magnetic characteristics were measured by the vibrating sample magnetometer (VSM) with a maximum applied field of 20 kOe at room temperature. All the measurements were focused on the Fe/Pt multilayer film structures without and with insertion of two ultrathin Ag nanolayers, which was done in order to explore the perpendicular magnetization reversal mechanism of the FePt system with the insertion of Ag nanolayers accompanied by continuous to nanogranular microstructure types.

## 3. Results and Discussion

**Figure 1:**
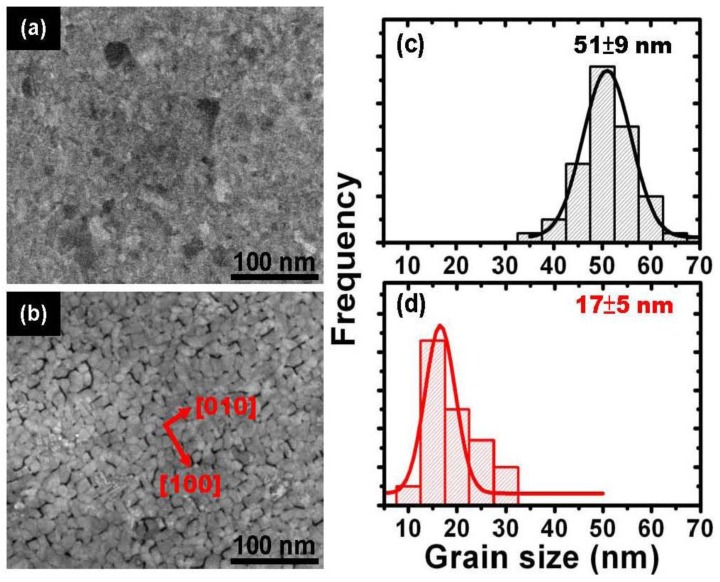
Field emission scanning electron microscope (FE-SEM) surface morphology images for the [Fe/Pt]_18_ multilayer films (**a**) without and (**b**) with two ultrathin Ag (0.5 nm each) nanolayers. The corresponding grain size histograms for evaluating average size and distribution of the FePt films (**c**) without and (**d**) with insertion of two Ag nanolayers are related to (**a**) and (**b**), respectively.

Figure 1 shows the FE-SEM images for the [Fe/Pt]_18_ multilayer films (Figure 1a) without and (Figure 1b) with the insertion of two ultrathin Ag (0.5 nm each) nanolayers, respectively. The grain size histograms for evaluating the average size and distribution of the FePt films without and with the insertion of Ag nanolayers are shown in Figure 1c,d corresponding to Figure 1a,b, respectively. A clear change in the surface morphology of the FePt films is observed and modified with the insertion of Ag nanolayers, and the average grain size of the FePt decreases with the insertion of Ag. The grains of the FePt film without the insertion of Ag are connected with each other, and the structure appears like a continuous film, as shown in Figure 1a. On the other hand, the surface morphology transforms from continuous to nanogranular structures in the FePt with the insertion of Ag nanolayers as shown in Figure 1b. It indicates that the continuous growth of the pure FePt film is interrupted by the insertion of the Ag nanolayers and results in the formation of nanogranular FePt grains with (001) preferred crystallographic orientation. FePt *L*1_0_ anisotropic rectangular-like nanogranular grains with the insertion of Ag are aligned along the direction of MgO[100] with (001) preferred orientation, and the major facet planes are (100) and (010), as shown in Figure 1b. The nanograin boundaries of FePt-Ag domain particles will work as functional pinning sites to enhance coercivity effectively. The grains in the fraction in Figure 1a range in size from 42 to 60 nm, with an average size of 51 ± 9 nm, as shown in Figure 1c; the grains in the fraction in Figure 1b contain grains ranging in size from 12 to 22 nm, with an average size of 17 ± 5 nm, as shown in Figure 1d. The grain size histograms for fractions are shown in Figure 1c,d and confirm a significant degree of FePt size reduction with the insertion of Ag nanolayers. The FePt–Ag nanocomposite films with nanogranular microstructure composed partial single-domain particles with sizes of tens of nanometers. Hence, the magnetization reversal process could be relative closer to the coherent rotation. The pure FePt film with a continuous microstructure will belong to the magnetic domain wall displacement; in other words, it is a closer multidomain structure.

**Figure 2:**
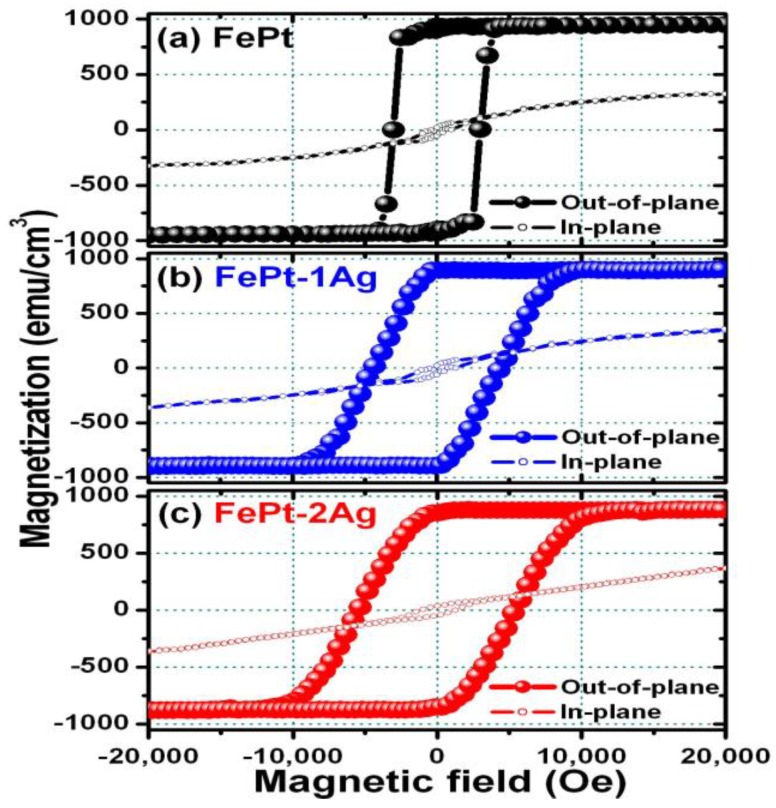
Out-of-plane and in-plane magnetization curves for the [Fe/Pt]_18_ multilayer films (**a**) without and with insertion of (**b**) a single and (**c**) two ultrathin Ag (0.5 nm each) nanolayers denoted as FePt–1Ag and FePt–2Ag, respectively. The magnetic field was applied in the out-of-plane direction (full symbols, ●) and in the in-plane direction (open symbols, o) to the film, respectively.

Figure 2 shows the out-of-plane (full symbols) and in-plane (open symbols) magnetization curves for the [Fe/Pt]_18_ multilayer films (Figure 2a) without and with the insertion of (Figure 2b) a single and (Figure 2c) two ultrathin Ag (0.5 nm each) nanolayers, respectively. Compared with the out-of-plane and in-plane magnetization curves, it is clear shown that the out-of-plane direction is the magnetic easy axis. The perpendicular anisotropy manifests for all FePt films without and with the insertion of ultrathin Ag nanolayers. The out-of-plane coercivity, magnetic squareness, saturation magnetization, and uniaxial magnetic anisotropy energy (*K_u_*) values for FePt films without and with the insertion of ultrathin Ag nanolayers are apparently different. The clear Ag insertion effects and their corresponding magnetic data are listed in Table 1. The perpendicular magnetization reversal behavior and the microstructure effect of FePt films without and with the insertion of ultrathin Ag nanolayers will be investigated and discussed below.

**Figure 3:**
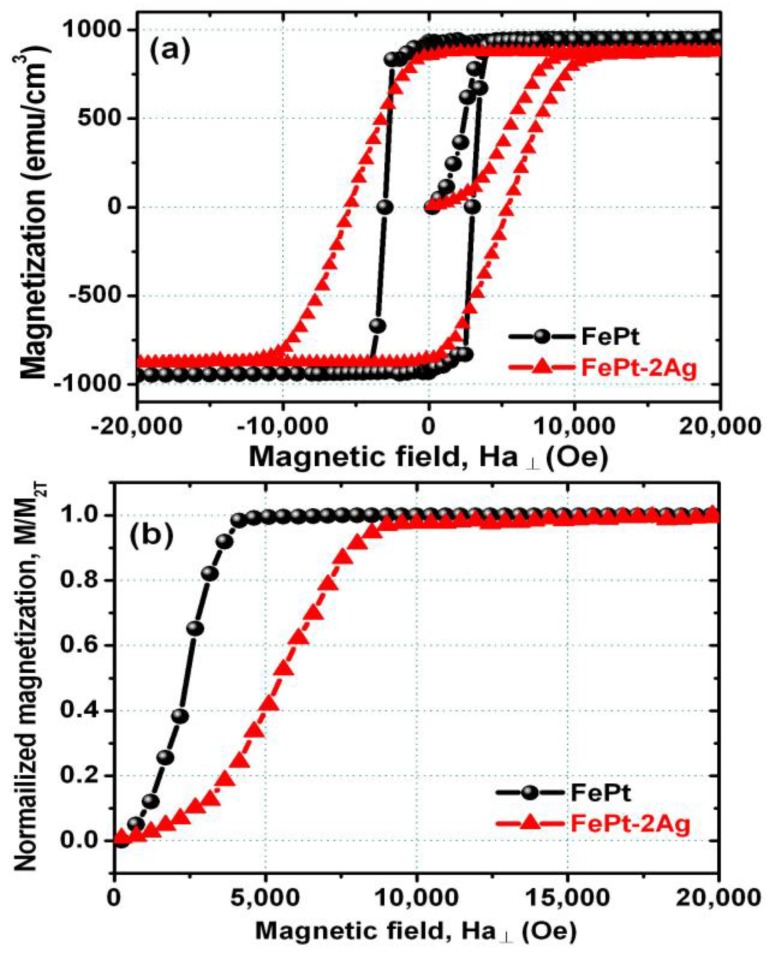
(**a**) Out-of-plane magnetization curves and corresponding (**b**) normalized initial magnetization curves for the [Fe/Pt]_18_ multilayer films without and with insertion of two ultrathin Ag (0.5 nm each) nanolayers.

The obvious variation of the magnetization processes in FePt films without and with the insertion of two ultrathin Ag nanolayers is clearly demonstrated by the initial magnetization curves, as shown in Figure 3. The out-of-plane coercivity of the pure FePt shows a value of 2998 Oe (without Ag), as shown in Figure 3a and listed in Table 1. It is clearly shown that with the insertion of a single and two ultrathin Ag nanolayers into 18 nm FePt multilayer films, the out-of-plane coercivity increases to 4220 and 5264 Oe, respectively. The out-of-plane saturation magnetization (*M_s_***_﬩_**) value decreased with the insertion of a single and two Ag nanolayers into the FePt system, ranging from 950 to 900 emu/cm^3^ and 875 emu/cm^3^, and the remanent magnetization (*M_r_***_﬩_**) also decreased from 932 to 876 emu/cm^3^ and 835 emu/cm^3^ following the slope of *M_s_*. This result can be understood as Ag is a non-magnetic element, and it slightly dilutes the magnetization of the FePt films. The uniaxial magnetic anisotropy energy (*K_u_*) also slightly decreased from 2.5 × 10^7^ to 2.3 × 10^7^ erg/cm^3^ and 2.1 × 10^7^ erg/cm^3^ with the FePt insertion of single and two Ag nanolayers, which was due to the crystallographic texture and misalignment of the anisotropy axis of the FePt changing slightly due to the insertion of Ag. The out-of-plane remanent squareness ratio (*M_r_***_﬩_**/*M_s_***_﬩_**) for the pure FePt was close to unity (0.98), and decreased to 0.97 and 0.95 with the insertion of a single and two Ag nanolayers into the FePt system, which implied the decrease of the exchange coupling of the FePt system with the insertion of Ag nanolayers. The normalized initial magnetization curve will be used to elucidate the magnetization reversal mechanism, as shown in Figure 3b. For the FePt films with the insertion of Ag nanolayers, they became much harder to saturate compared the pure FePt films at the same applied magnetic field. It could be understood if the magnetization reversal behavior was dominated by pinning sites, and the domain wall movement would not move unless the external applied magnetic field was larger than the pinning field. If the magnetization reversal behavior is close to the rotation of the Stoner-Wohlfarth (S-W) mode, the single domain grains only reversed their magnetization behavior when the external applied magnetic field exceeded the anisotropy energy [49,50,51,52,53]. Thus, the pure FePt film without the insertion of Ag nanolayers shows a close to nucleation type of initial magnetization curve, and FePt film with the insertion of Ag nanolayers shows a close to typical pinning type of initial magnetization curve.

**Figure 4:**
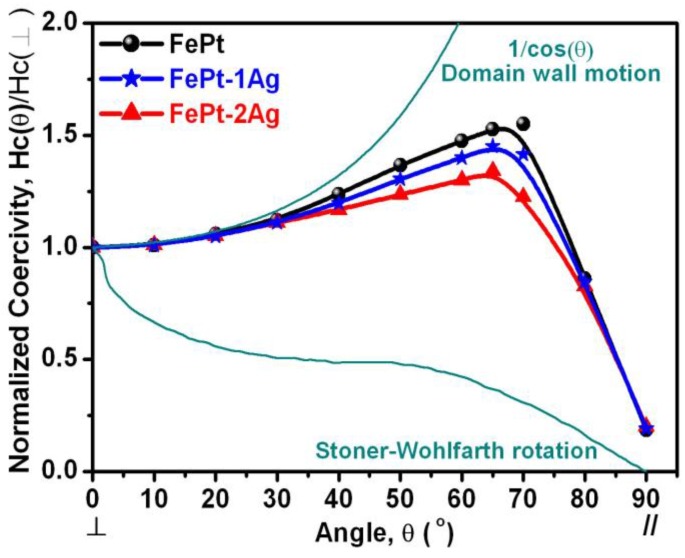
Angular dependence of coercivity for the [Fe/Pt]_18_ multilayer films without and with insertion of a single and two ultrathin Ag (0.5 nm each) nanolayers. The angle is referred to that between the easy axis (film normal) and the applied field direction.

For the purpose of understanding the magnetization reversal behavior of the FePt films without and with ultrathin Ag nanolayer dependence, the angular dependence of coercivity for the FePt multilayer films without and with the insertion of a single and two Ag nanolayers was explored, as shown in Figure 4. The ideally theoretical Kondorsky (incoherent switching by domain wall motion in highly exchange coupled systems) and Stoner-Wohlfarth (S-W, coherent rotation for isolated particles) models are two boundary conditions also shown in Figure 4. The angular dependence of the coercivity profile for the FePt film without the insertion of Ag shows a typical peak behavior, which is closer to the domain-wall-motion mode; in other words, it is closer to multidomain structures. When the FePt films were inserted with a single and two Ag nanolayers, the profiles were more similar to coherent S-W rotation and less similar to domain-wall motion due to the Ag element which could decouple the inter-grain exchange coupling between the FePt neighboring grains. Thus, the magnetization reversal behavior of FePt–Ag nanocomposite films becomes more independently associated with incoherent rotation. The tendency is toward weakened domain-wall motion behavior, while an enhanced rotation mode is found in the perpendicular magnetization reversal process for the FePt–Ag nanocomposite system.

**Figure 5:**
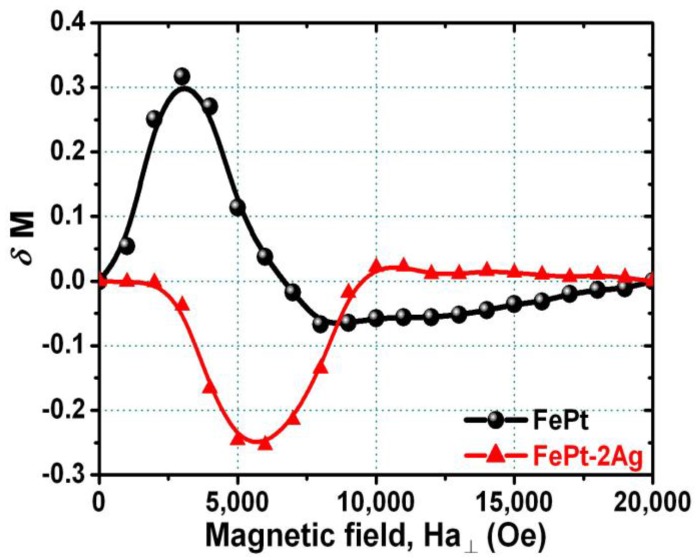
Kelly-Henkel (δ*M*) plot for the [Fe/Pt]_18_ multilayer films without and with insertion of two ultrathin Ag (0.5 nm each) nanolayers. The magnetic field was applied in the out-of-plane direction to the film.

Figure 5 shows the Kelly-Henkel plot (δ*M* measurement) for FePt films without and with the insertion of two ultrathin Ag nanolayers, respectively. The δ*M* measured mode is used to determine the inter-grain interaction in many hard magnetic materials, which is defined as [54,55,56]:

δ*M* = *M_DCD_*(*H*) − [1 − 2*M_IRM_*(*H*)] (1)

where *M_DCD_*(*H*) and *M_IRM_*(*H*) are the normalized dc-demagnetization remanence and isothermal remanence as a function of the external magnetic field, respectively. The positive δ*M* peak indicates ferromagnetic inter-grain interactions. On the other hand, the negative δ*M* peak exhibits dipole interactions associated with incoherent rotation. It can be seen from Figure 5 that FePt film without the insertion of Ag has a positive δ*M* (strong ferromagnetic interaction), whereas FePt film with the insertion of Ag nanolayers has a negative δ*M* at all applied magnetic fields (dipole interaction). The parameter δ*M* value is well known to lead media noise, and this value of δ*M* can be simply controlled by the insertion of Ag nanolayers into the FePt system in this work. The reduction of exchange interactions between neighboring grains in the FePt films has been achieved by controlling the insertion of Ag nanolayers, and a lower value of δ*M* is desirable. The above results suggested that the microstructure and the magnetization reversal mechanism including inter-grain exchange coupling of the FePt(001) films could be tuned and affected by the insertion of Ag nanolayers into the Fe/Pt multilayers. This is due to the microstructural modification of FePt films promoted by the insertion of Ag nanolayers even at a low processing temperature of 300 °C, so the inter-grain exchange coupling weakened and the corresponding magnetic reversal mechanism changed.

**Figure 6:**
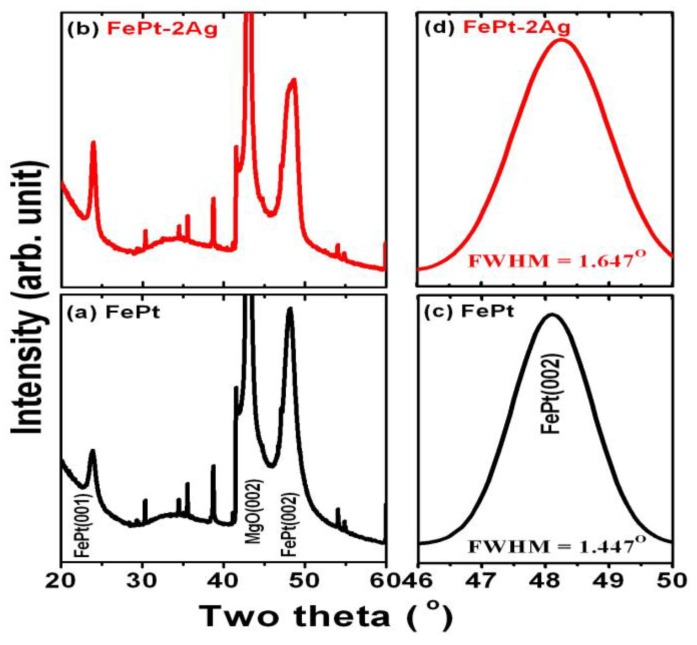
X-ray diffraction scan patterns (XRD) for the [Fe/Pt]_18_ multilayer films (**a**) without and (**b**) with two ultrathin Ag (0.5 nm each) nanolayers. The corresponding slow scan curves of the FePt (002) peak in the θ–2θ scan (**c**) without and (**d**) with insertion of two Ag nanolayers are related with (**a**) and (**b**), respectively.

Figure 6 shows the X-ray diffraction patterns for the [Fe/Pt]_18_ multilayer films (Figure 6a) without and (Figure 6b) with the insertion of two ultrathin Ag nanolayers directly evaporated onto MgO(100) substrates, respectively.

The slow scan curves of the FePt(002) diffraction peak in the θ–2θ scan as shown in Figure 6c,d correspond to Figure 6a,b, respectively. In addition to the fundamental (002) peak, the (001) superlattice peak of the *L*1_0_-ordered FePt phase has been clearly observed for both films. The unlabeled sharp peaks are due to the MgO(001) single-crystal substrate. In Figure 6, no peaks except the (00*n*) diffraction peaks are observed in the whole diffraction patterns (θ–2θ scan), indicating the Fe/Pt multilayer film structures without and with the insertion of Ag nanolayers are strongly textured to the (001) planes, and this also confirms the structures did epitaxially grow onto the MgO substrates. The FePt peaks are relatively broad for the FePt films with the insertion of Ag nanolayers compared to the FePt without Ag. Additionally, the full width at half maximum (FWHM) value of the slow scan curves of FePt(002) for FePt with the insertion of Ag (1.647°) is larger than that of pure FePt without Ag (1.447°), as shown in Figure 6c,d. According to the results mentioned above, which indicate the grain size of FePt films is decreased with the insertion of Ag nanolayers into the FePt films, this fact is consistent with the FE-SEM observation.

## 4. Conclusions

The perpendicular magnetization reversal behavior and the exchange-coupling interaction of the low-temperature-ordered FePt films without and with the insertion of Ag nanolayers have been successfully demonstrated. The grain size and corresponding inter-grain interaction of the FePt alloy films can be reduced by the insertion of ultrathin Ag nanolayers into the FePt system. The most notable effect of the insertion of Ag nanolayers into FePt is that the surface topography changes from continuous to nanogranular microstructures. The coercivity improvement is mainly due to the formation of nanogranular FePt grains induced by the insertion of Ag nanolayers. The change in the magnetization process from less similar to domain wall displacement to more similar to the rotation mode with the insertion of Ag nanolayers into the FePt system was clearly demonstrated as the surface topography varied from continuous to nanogranular microstructures. This research work could provide a simple method for fabricating nanogranular FePt(001) films via the insertion of Ag nanolayers and with tunable related magnetization reversal behavior in general at a relatively low processing temperature of 300 °C in the potential industrial development for ultrahigh-density perpendicular spin electronic devices and information storage medium applications.

## Figures and Tables

**Table 1:** Out-of-plane coercivity (*H_c_***_﬩_**) saturation magnetization (*M_s_***_﬩_**), remanent magnetization (*M_r_***_﬩_**), remanent squareness ratio (*M*_r_**_﬩_**/*M_s_***_﬩_**), and uniaxial magnetic anisotropy energy (*K_u_*) values for the [Fe/Pt]_18_ multilayer films without and with the insertion of a single and two ultrathin Ag (0.5 nm each) nanolayers.

Structures (Denoted)	Film Structures (Thickness nm)	*H_c_*_﬩_ (Oe)	*M_s_*_﬩_ (emu/m^3^)	*M*_r__﬩_ (emu/m^3^)	*M*_r__﬩_/*M_s_*_﬩_ (ratio)	*K_u_* (erg/m^3^)
FePt	[Fe/Pt]_18_	2998	950	932	0.98	2.5 × 10^7^
FePt–1Ag	[Fe/Pt]_9_/Ag_0.5_/[Fe/Pt]_9_	4220	900	876	0.97	2.3 × 10^7^
FePt–2Ag	[Fe/Pt]_6_/Ag_0.5_/[Fe/Pt]_6_/Ag_0.5_/[Fe/Pt]_6_	5264	875	835	0.95	2.1 × 10^7^

## References

[1] Weller D., Moser A., Folks L., Best M.E., Lee W., Toney M.F., Schwickert M., Thiele J.U., Doerner M.F. (2000). High *K_u_* materials approach to 100 Gbits/in^2^. IEEE Trans. Magn..

[2] Skomski R. (2003). Nanomagnetics. J. Phys.: Condens. Matter.

[3] Chappert C., Fert A., van Dau F.N. (2007). The emergence of spin electronics in data storage. Nature Mater..

[4] Seki T., Hasegawa Y., Mitani S., Takahashi S., Imamura H., Maekawa S., Nitta J., Takanashi K. (2008). Giant spin Hall effect in perpendicularly spin-polarized FePt/Au devices. Nature Mater..

[5] Matsumoto S., Shima T. (2011). Magnetic properties of FePt thin films with multilayered structure. J. Phys. Conf. Ser..

[6] Ho P., Han G.C., Chow G.M., Chen J.S. (2011). Interlayer magnetic coupling in perpendicular anisotropy *L*10-FePt based pseudo spin valve. Appl. Phys. Lett..

[7] Seki T., Hotta K., Imamura H., Nozaki Y., Takanashi K. (2013). Characteristic field angular dependence of magnetization switching assisted by spin wave excitation. Appl. Phys. Lett..

[8] Seki T., Utsumiya K., Nozaki Y., Imamura H., Takanashi K. (2013). Spin wave-assisted reduction in switching field of highly coercive iron-platinum magnets. Nature Commun..

[9] Pal S., Barman S., Hellwig O., Barman A. (2014). Effect of the spin-twist structure on the spin-wave dynamics in Fe_55_Pt_45_/Ni_80_Fe_20_ exchange coupled bi-layers with varying Ni_80_Fe_20_ thickness. J. Appl. Phys..

[10] Hussain S., Bhatia C.S., Yang H., Danner A.J. (2014). Effect of FePt on resonant behaviour of a near field transducer for high areal density heat assisted magnetic recording. Appl. Phys. Lett..

[11] Seki T., Yako H., Yamamoto T., Kubota T., Sakuraba Y., Ueda M., Takanashi K. (2015). Spin torque-induced magnetization dynamics in giant magnetoresistance devices with Heusler alloy layers. J. Phys. D: Appl. Phys..

[12] Luo C.P., Liou S.H., Gao L., Liu Y., Sellmyer D.J. (2000). Nanostructured FePt:B_2_O_3_ thin films with perpendicular magnetic anisotropy. Appl. Phys. Lett..

[13] Hsu Y.N., Jeong S., Laughlin D.E., Lambeth D.N. (2001). Effects of Ag underlayers on the microstructure and magnetic properties of epitaxial FePt thin films. J. Appl. Phys..

[14] Zhang Z.G., Kang K., Suzuki T. (2003). FePt (001) texture development on an Fe–Ta–C magnetic soft underlayer with SiO_2_/MgO as an intermediate layer. Appl. Phys. Lett..

[15] Kang K., Zhang Z.G., Papusoi C., Suzuki T. (2004). Composite nanogranular films of FePt-MgO with (001) orientation onto glass substrates. Appl. Phys. Lett..

[16] Peng Y., Zhu J.G., Laughlin D.E. (2006). *L*10 FePt–MgO perpendicular thin film deposited by alternating sputtering at elevated temperature. J. Appl. Phys..

[17] Li B.H., Feng C., Gao X., Teng J., Yu G.H., Xing X., Liu Z.Y. (2007). Magnetic properties and microstructure of FePt/BN nanocomposite films with perpendicular magnetic anisotropy. Appl. Phys. Lett..

[18] Wei D.H., Yuan F.T., Chang H.W., Yao Y.D. (2008). Effects of Pt and Fe underlayers on the microstructure and magnetization reversal of epitaxial FePt films for high areal density magnetic recording. J. Appl. Phys..

[19] Feng C., Zhan Q., Li B.H., Teng J., Li M.H., Jiang Y., Yu G.H. (2008). Magnetic properties and microstructure of FePt/Au multilayers with high perpendicular magnetocrystalline anisotropy. Appl. Phys. Lett..

[20] Wei D.H., Yao Y.D. (2009). Controlling microstructure and magnetization process of FePd (001) films by staged thermal modification. Appl. Phys. Lett..

[21] Wei D.H. (2009). Magnetic assembles of FePt (001) nanoparticles with SiO_2_ addition. J. Appl. Phys..

[22] Kent A.D. (2010). Spintronics: Perpendicular all the way. Nature Mater..

[23] Ohsugi R., Kohda M., Seki T., Ohtsu A., Mizuguchi M., Takanashi K., Nitta J. (2012). MgO layer thickness dependence of structure and magnetic properties of *L*1_0_-FePt/MgO/GaAs structures. Jpn. J. Appl. Phys..

[24] Cuadrado R., Chantrell R.W. (2015). Interaction potential of FePt with the MgO(001) surface. Phys. Rev. B.

[25] Speliotis Th., Varvaro G., Testa A.M., Giannopoulos G., Agostinelli E., Li W., Hadjipanayis G., Niarchos D. (2015). Microstructure and magnetic properties of (001) textured *L*1_0_ FePt films on amorphous glass substrate. Appl. Surf. Sci..

[26] Endo Y., Kikuchi N., Kitakami O., Shimada Y. (2001). Lowering of ordering temperature for fct Fe–Pt in Fe/Pt multilayers. J. Appl. Phys..

[27] Shima T., Moriguchi T., Mitani S., Takanashi K. (2002). Low-temperature fabrication of *L*1_0_ ordered FePt alloy by alternate monatomic layer deposition. Appl. Phys. Lett..

[28] Chou S.C., Yu C.C., Liou Y., Yao Y.D., Wei D.H., Chin T.S., Tai M.F. (2004). Annealing effect on the Fe/Pt multilayers grown on Al_2_O_3_ (0001) substrates. J. Appl. Phys..

[29] Yao B., Coffey K.R. (2008). The effective interdiffusivity, structure, and magnetic properties of [Fe/Pt]*_n_* multilayer films. J. Appl. Phys..

[30] Yao B., Coffey K.R. (2009). Quantification of *L*1_0_ phase volume fraction in annealed [Fe/Pt]*_n_* multilayer films. J. Appl. Phys..

[31] Tanaka M., Ogata Y., Nakagawa S. (2011). FePt films 2 nanometers thick with (001) preferential orientation on a MgO underlayer. J. Appl. Phys..

[32] Wei D.H., Chi P.W., Chao C.H. (2014). Perpendicular magnetization reversal mechanism of functional FePt films for magnetic storage medium. Jpn. J. Appl. Phys..

[33] Hsu Y.N., Jeong S., Laughlin D.E., Lambeth D.N. (2003). The effects of Ag underlayer and Pt intermediate layers on the microstructure and magnetic properties of epitaxial FePt thin films. J. Magn. Magn. Mater..

[34] Zhao Z.L., Ding J., Inaba K., Chen J.S., Wang J.P. (2003). Promotion of *L*1_0_ ordered phase transformation by the Ag top layer on FePt thin films. Appl. Phys. Lett..

[35] Shao Y., Yan M.L., Sellmyer D.J. (2003). Effects of rapid thermal annealing on nanostructure, texture and magnetic properties of granular FePt:Ag films for perpendicular recording. J. Appl. Phys..

[36] Chen J.S., Hu J.F., Lim B.C., Phyoe W.L., Liu B., Ju G. (2009). Structure and magnetic properties of *L*1_0_ FePt film with Ag heat sink layer. J. Appl. Phys..

[37] Tsai J.L., Tzeng H.T., Lin G.B., Liu B.F. (2010). The Ag effect on magnetic properties and microstructure of FePt/Ag_2_Te particulate films. J. Alloy Compd..

[38] Wen W.C., Chepulskii R.V., Wang L.W., Curtarolo S., Lai C.H. (2012). Accelerating disorder–order transitions of FePt by performing a metastable AgPt phase. Acta Mater..

[39] Katona G.L., Vladymyrskyi I.A., Makogon I.M., Sidorenko S.I., Kristály F., Daróczi L., Csik A., Liebig A., Beddies G., Albrecht M. (2013). Grain boundary diffusion induced reaction layer formation in Fe/Pt thin films. Appl. Phys. A.

[40] Crisan A.D., Vasiliu F., Mercioniu I., Crisan O. (2014). Role of Ag addition in *L*1_0_ ordering of FePt-based nanocomposite magnets. Philosoph. Mag..

[41] Varaprasad B.S.D.Ch.S., Takahashi Y.K., Wang J., Ina T., Nakamura T., Ueno W., Nitta K., Uruga T., Hono K. (2014). Mechanism of coercivity enhancement by Ag addition in FePt-C granular films for heat assisted magnetic recording media. Appl. Phys. Lett..

[42] Kang K., Zhang Z.G., Papusoi C., Suzuki T. (2003). (001) oriented FePt–Ag composite nanogranular films on amorphous substrate. Appl. Phys. Lett..

[43] Wan J., Huang Y., Zhang Y., Bonder M.J., Hadjipanayis G.C., Weller D. (2005). Particulate FePt/Ag(C) films with strong perpendicular anisotropy. J. Appl. Phys..

[44] Zhao Z.L., Chen J.S., Ding J., Yi J.B., Liu B.H., Wang J.P. (2006). Fabrication and microstructure of high coercivity FePt thin films at 400 °C. Appl. Phys. Lett..

[45] Li B.H., Feng C., Yang T., Hwang P., Teng J., Yu G.H., Zhu F.W. (2006). Approach to enhance the coercivity in perpendicular FePt/Ag nanoparticle film. J. Appl. Phys..

[46] Chen J.S., Zhou Y.Z., Sun C.J., Han S.W., Chow G.M. (2011). Where is the Ag in FePt-Ag composited films?. Appl. Phys. Lett..

[47] Takahashi Y.K., Hono K., Shima T., Takanashi K. (2003). Microstructure and magnetic properties of FePt thin films epitaxially grown on MgO (001) substrates. J. Magn. Magn. Mater..

[48] Liu M., Jin T., Hao L., Cao J., Wang Y., Wu D., Bai J., Wei F. (2015). Effects of Ru and Ag cap layers on microstructure and magnetic properties of FePt ultrathin films. Nanoscale Res. Lett..

[49] Wohlfarth E.P. (1984). The coefficient of magnetic viscosity. J. Phys. F: Met. Phys..

[50] Gau J.S., Brucker C.F. (1985). Angular variation of the coercivity in magnetic recording thin films. J. Appl. Phys..

[51] Byun C., Sivertsen J.M., Judy J.H. (1986). A study on magnetization reversal mechanisms of CoCr films. IEEE Trans. Magn..

[52] Suzuki T., Honda N., Ouchi K. (1999). Magnetization reversal process in polycrystalline ordered Fe–Pt(001) thin films. J. Appl. Phys..

[53] Coffey K.R., Thomson T., Thiele J.U. (2003). Angle dependent magnetization reversal of thin film magnetic recording media. J. Appl. Phys..

[54] Ma Y.G., Yang Z., Matsumoto M., Morisako A., Takei S. (2003). Structural and magnetic properties of NdFeB thin films sputtered on W underlayers. J. Magn. Magn. Mater..

[55] Liu X.X., Ishida G., Morisako A. (2011). Magnetization reversal mechanism of Nd–Fe–B films with perpendicular magnetic anisotropy. J. Appl. Phys..

[56] Kelly P.E., O’Grady K., Mayo P.I., Chantrell R.W. (1989). Switching mechanisms in cobalt-phosphorus thin films. IEEE Trans. Magn..

